# Novel reporter systems to detect cold and osmotic stress responses

**DOI:** 10.1093/biomethods/bpaf048

**Published:** 2025-06-14

**Authors:** Kanon Maruyama, Hodaka Fujii

**Affiliations:** Department of Biochemistry and Genome Biology, Hirosaki University Graduate School of Medicine, 5 Zaifu-cho, Hirosaki, Aomori 036-8562, Japan; Department of Biochemistry and Genome Biology, Hirosaki University Graduate School of Medicine, 5 Zaifu-cho, Hirosaki, Aomori 036-8562, Japan

**Keywords:** reporter, cold stress, osmotic stress, inducible translocation trap (ITT)

## Abstract

Cells respond to environmental stresses such as cold and osmotic stresses. These stresses induce signal transduction pathways in cells. However, the molecular mechanisms activated by cold and osmotic stresses in higher eukaryotes remain elusive. Previously, we described a reporter system utilizing inducible translocation trap that detects nuclear translocation of 2-amino-3-ketobutyrate coenzyme A ligase (KBL) in response to cold and osmotic stresses. In the present study, we developed additional reporter systems to detect intracellular events induced by these stresses. These reporter systems will be instrumental to elucidate the intracellular signaling mechanisms activated by these stresses.

## Introduction

If the body temperature of humans falls below 27°C, neuromuscular, cardiovascular, hematological, and respiratory changes can prove fatal [1]. When cells of humans and other mammals are exposed to cold stress, various mechanisms maintain cellular homeostasis, including those that control gene expression induced by moderate cold stress (25–33°C) in the nucleus [2]. In the transduction processes of peripheral thermal sensation, some members of the transient receptor potential (TRP) family play a critical role in cold perception by sensory neurons. TRP channels are expressed in cell membranes and membranes of internal structures. In particular, canonical transient receptor potential 5 (TRPC5) is expressed in 75% of human sensory neurons [3]. The roles of these channels have been reported and reviewed in detail [4–8]. The mechanisms underlying cold perception by other cell types have not been clarified. Phosphorylation of p38 and translocation of β-crystallin from the nucleus to the cytoplasm have been reported [9, 10]. Cold stress inhibits cell proliferation [11] and induces expression of inflammatory cytokines [2]. During cold shock, production of specific proteins is promoted, including cold-induced proteins (CIPs), cold acclimatization proteins (CAPs), and cold shock proteins (CSPs). CSPs can be both CIPs and CAPs and contain a conserved nucleic acid–binding domain [12, 13]. CSPs and their cold shock domains have been reviewed elsewhere [14].

Cold and osmotic stresses may induce signal transduction involving nuclear translocation of signaling molecules. We previously described a reporter system for cold and osmotic stresses [15] utilizing the inducible translocation trap (ITT) technology [16]. In ITT, a fusion protein of LexA DNA-binding domain (LexA DB), a transactivation (TA) domain of a transcription activator protein such as Gal4 and VP16, and a test protein is expressed in a reporter cell line harboring a reporter gene such as green fluorescent protein (GFP) or human CD2, which encodes a cell surface protein, together with LexA operator sequences. Nuclear translocation of the fusion protein induces binding of LexA DB to the LexA operator, which activates transcription of the reporter gene (Supplementary Fig. S1). ITT has been utilized to detect nuclear translocation of the M2 isoform of pyruvate kinase induced by growth signaling [17], an effector molecule of malaria parasites [18], and the cytoplasmic domain of a receptor of neurotrophic factors [19]. It has also been used to analyze regulation of STAT transcription factors by cytokines [20, 21]. We previously described a reporter system to detect cold and osmotic stresses that consists of the reporter genes and a fusion protein of LexA DB, a TA domain, and 2-amino-3-ketobutyrate coenzyme A ligase (KBL) [15].

In the present study, we developed two new reporter systems to detect signaling of cold and osmotic stresses. They consist of LexA DB, a TA domain, and a partial fragment of leucine-rich repeat-containing protein 45 (LRRC45) or coiled-coil domain-containing protein 91 (CCDC91). These reporter gene systems will be useful to analyze cold and osmotic stresses.

## Materials and methods

### ITT screening of stress-responsive proteins

ITT screening of stress-responsive proteins was performed as previously described [15]. Briefly, a cDNA library derived from mouse hematopoietic Ba/F3 cells [22] was constructed in the pLG vector expressing a fusion protein consisting of LexA DB, the TA domain of Gal4, and a protein encoded by a cDNA (LG-fusion protein) [16] and transduced into the Ba/F3-derived BLG cell line harboring the LexA-d1EGFP reporter gene. Library-transduced cells were sorted at room temperature for exposure to cold stress and high pressure, and the GFP (-) population was acquired. At 4 h after GFP (-) sorting, GFP (+) cells, which were induced to express GFP by exposure to cold stress and high pressure, were sorted. After incubation for several days to downregulate GFP expression, these cells were subjected to another round of GFP (-) and GFP (+) sorting. Subsequently, sorted cells were subjected to single-cell sorting. Each clone was exposed to cold or osmotic stress and then GFP expression was examined.

### Amplification of cDNA inserts and sequencing

Genomic DNA was prepared from clones that responded to cold and osmotic stresses and subjected to PCR amplification using viral vector primers [16]. The amplicons were cloned into the pLG vector and subjected to Sanger sequencing. The following pLG-fusion plasmids were deposited to and are available through Addgene: pLG-mLRRC45 (158 a.a.), Addgene Plasmid #240067; pLG-mLRRC45 (209 a.a.), Addgene Plasmid #240068; and pLG-mCCDC91 (198 a.a.), Addgene Plasmid #240069.

### Re-transduction assay

To confirm that fusion proteins of LG and proteins or protein fragments encoded by the cDNA inserts responded to cold and osmotic stresses, BLG cells were transduced with the LG-fusion retroviral plasmids, exposed to cold or osmotic stress, and analyzed by flow cytometry to monitor GFP expression levels.

## Results and discussion

**Figure 1:**
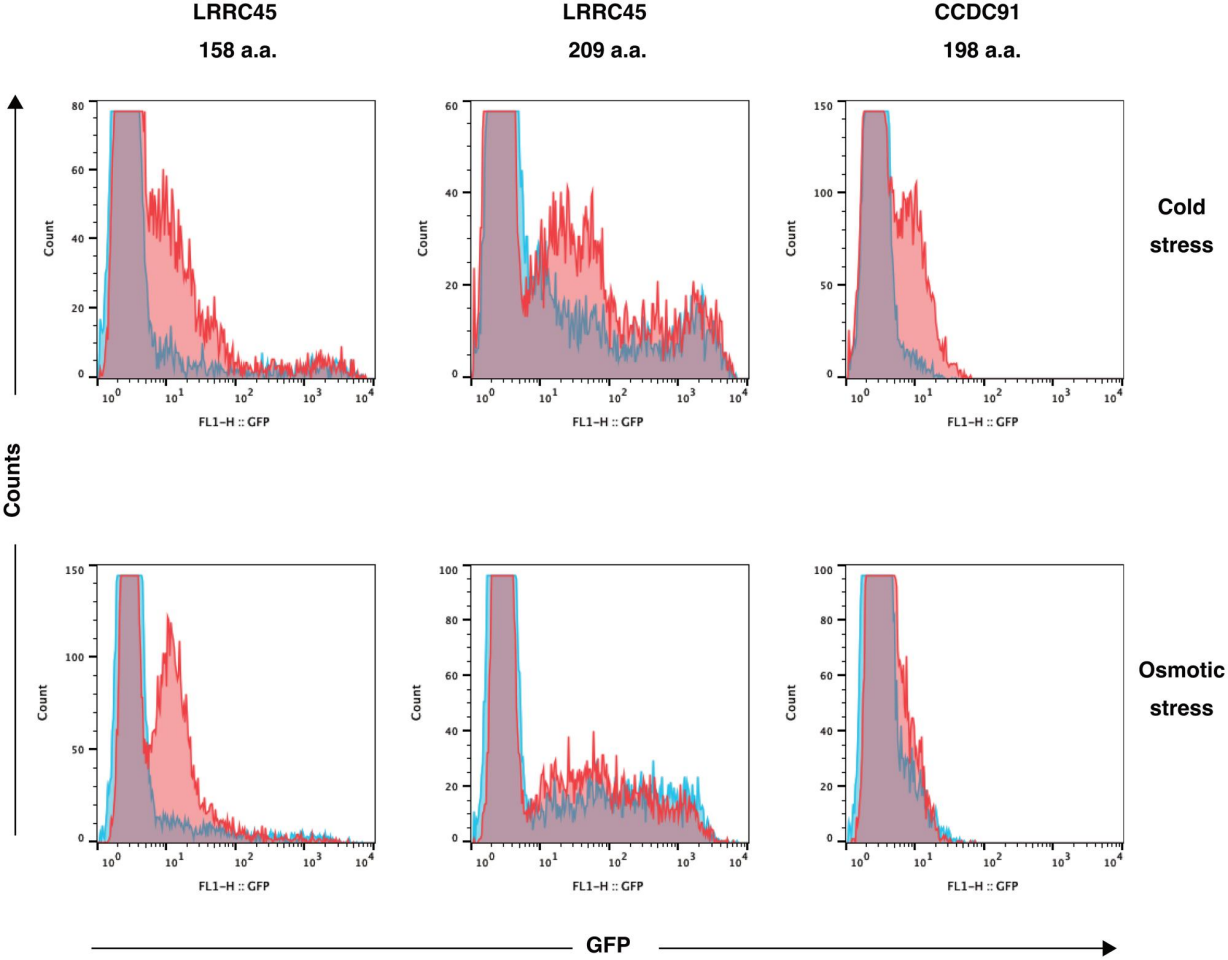
Induction of reporter gene expression by cold and osmotic stresses cDNA inserts were amplified by genomic PCR and subcloned into the pLG vector. The resultant plasmids expressing LG-fusion proteins were transduced into BLG cells. Transduced cells were subjected to cold stress (25°C for 30 min, upper panels) or osmotic stress (0.5 M sorbitol, lower panels). After 4 h, GFP expression was measured by flow cytometry. Not all cells expressed GFP in response to stress because the transduction efficiency was not 100%. Blue: mock stimulation; red: cold or osmotic stress.

Previously, we performed ITT screening to obtain a reporter system consisting of a LexA operator: the reporter fusion gene and the LG-KBL fusion protein [15]. We performed a similar screening to obtain two additional reporter systems (Supplementary Fig. S2). Briefly, a cDNA library expressing LG-fusion proteins was transduced into the BLG reporter cell line harboring the LexA-d1EGFP reporter gene. Subsequently, transduced cells were exposed to cold stress and high pressure, and GFP (+) cells were sorted after several hours. After several rounds of flow cytometric sorting of GFP (-) cells in the absence of cold stress to reduce background and GFP (+) cells after cold stress, single cells were cloned to perform genomic PCR of transduced retroviruses. Amplified cDNA inserts were subcloned into the pLG vector, and each resultant retroviral construct was transduced into BLG cells to examine whether cold and osmotic stresses induced reporter expression. Stress-induced reporter gene expression was observed with cDNA fragments of two genes (Fig. 1).

### LRRC45

**Figure 2:**
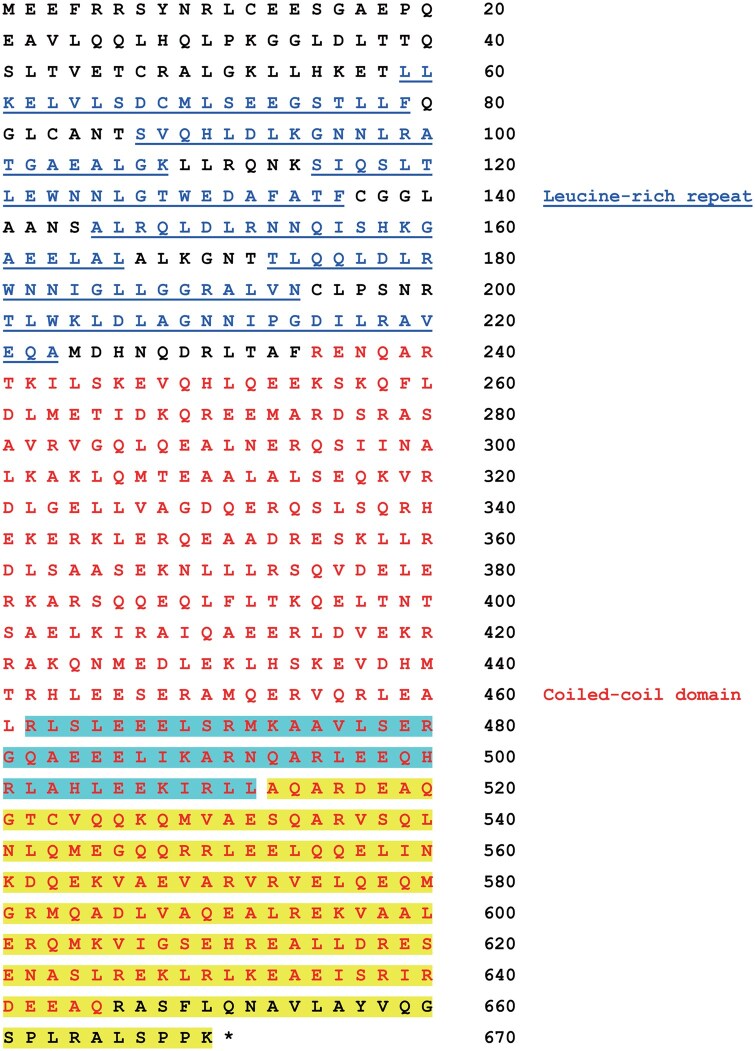
The a.a. sequence of LRRC45 LRRC45 consists of 670 a.a. residues. LG was fused with the C-terminal 158 (highlighted in yellow) or 209 (highlighted in cyan and yellow) a.a. residues. The leucine-rich repeats are shown in blue and underlined. The coiled-coil domain is shown in red.

The first was LG fused with the C-terminal 209 or 158 amino acid (a.a.) residues of LRRC45 (https://www.uniprot.org/uniprotkb/Q8CIM1/entry, Fig. 2). The full-length protein contains 670 a.a. residues, in which six leucine-rich repeats and one coiled-coil domain were identified (Fig. 2). The shorter fusion protein (the C-terminal 158 a.a. of LRRC45, highlighted in yellow in Fig. 2) markedly induced expression of the reporter gene in response to both cold and osmotic stresses (Fig. 1, left panel). By contrast, the longer fusion protein (the C-terminal 209 a.a. of LRRC45, highlighted in blue and yellow in Fig. 2) responded to cold stress but not osmotic stress (Fig. 1, middle panel). In addition, the longer fusion protein induced higher background expression of the reporter (Fig. 1, middle panel). This result suggests that the signal transduction mechanisms activated by cold and osmotic stresses share common components but also have specific components. The region only present in the longer fusion protein may contain a constitutive nuclear localization signal (NLS), although an obvious NLS sequence was not identified in this region.

LRRC45 is involved in centrosome cohesion as part of the linker that holds together the duplicated centrosome [23–27]. It is present in the centrosome, cytosol, and nucleoplasm [24], is recruited to the proximal end of the centriole by C-Nap1 [24], and is phosphorylated by NEK2 during mitosis [24], which decreases its centrosomal localization and ultimately causes centriole separation [24]. LRRC45 is also directly involved in formation of cilia in association with the distal mother centriole, Cep83, and SCLT1 [28]. Variants of LRRC45 cause ciliopathies [28]. LRRC45 may promote growth, metastasis, and invasion of lung cancer cells by increasing expression of c-MYC, Slug, MMP2, and MMP9 [29].

### CCDC91

**Figure 3:**
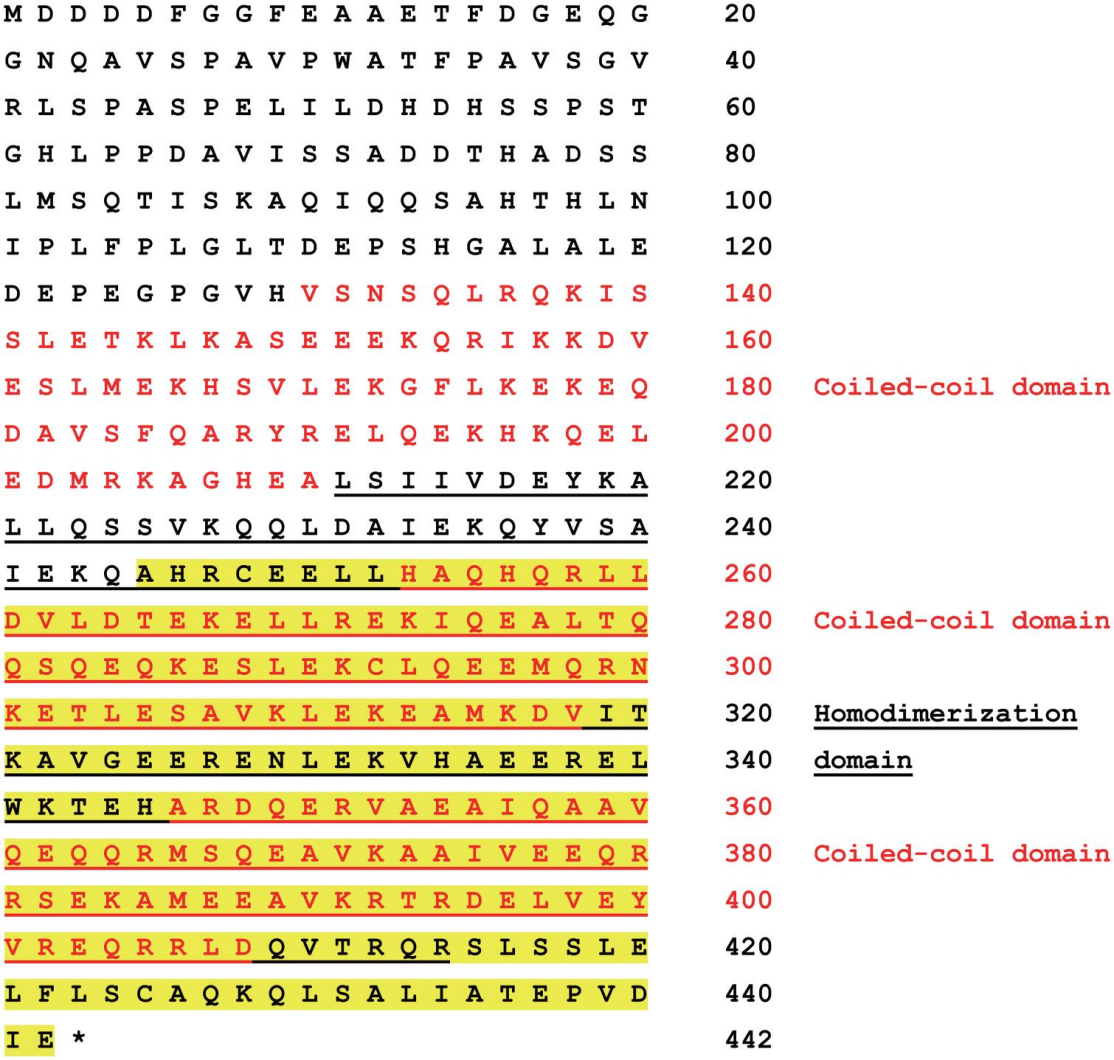
The a.a. sequence of CCDC91 CCDC91 consists of 442 a.a. residues. LG was fused with the C-terminal 198 a.a. residues (highlighted in yellow). The coiled-coil domains are shown in red. The homodimerization domain is underlined.

The second was LG fused with the C-terminal 198 a.a. residues of CCDC91 (https://www.uniprot.org/uniprotkb/Q9D8L5/entry, Fig. 3). The full-length protein contains 442 a.a. residues, in which three coiled-coil domains were identified (Fig. 3). The fusion protein clearly increased reporter gene expression in response to cold stress but only marginally in response to osmotic stress (Fig. 1, right panel). This result also suggests that the responses to cold and osmotic stresses have distinct features.

CCDC91, also known as p56, regulates membrane trafficking through the trans-Golgi network by cooperating with Golgi-localized, γ-ear containing, ADP-ribosylation factor-binding proteins (GGAs) during sorting of hydrolase to lysosomes [30–32].

CCDC91 is involved in bone remodeling. It plays an important role in elastin transport [33]. CCDC91 has also been suggested to promote proliferation and differentiation of myoblasts to mitigate skeletal muscle atrophy by regulating expression of insulin receptor substrates and activating a transduction pathway in chickens [34]. It is also involved in progression of mechanical stress-induced ossification [35].

Its encoding gene appears to be involved in the genetic predisposition to hand osteoarthritis. It is also involved in development of non-Hodgkin’s lymphoma [36]. CCDC91 has also been suggested to be associated with sperm teratology and azoospermia [37]. It is strongly expressed in tumor tissues of luminal-type breast cancer patients at risk of early recurrence [38, 39]. CCDC91 is also associated with variants that are related to breast cancer risk [40]. It has been suggested to be involved in the pathological mechanisms of bipolar disorder and treatment-resistant schizophrenia [41]. CCDC91 has been suggested to be one of the genes that is expressed specifically in COVID-19 patients and has been implicated in lung function [42, 43].

## Conclusions

Here, we developed ITT-based novel reporter systems to detect cold and/or osmotic stresses. These reporter systems will be useful to analyze intracellular signaling mechanisms activated by these stresses. LG-KBL reported previously [15] and pLG-mLRRC45 (158 a.a.) responded to both cold and osmotic stresses and thereby induced expression of the GFP reporter in BLG cells. By contrast, pLG-mLRRC45 (209 a.a.) and pLG-mCCDC91 (198 a.a.) responded to cold stress but only marginally responded to osmotic stress. These reporter systems will provide a unique opportunity to elucidate the specific signaling mechanisms activated by cold and osmotic stresses. For example, genetic screening methods such as clustered regularly interspaced short palindromic repeats (CRISPR) screening [44] using these reporters could identify potential signal transduction molecules that are differentially involved in these stress-sensing pathways.

## Limitations

Although we showed that the reporter systems can detect cold and/or osmotic stresses, it is unknown how these stresses activate these systems. In this regard, genetic methods such as CRISPR screening using these reporter systems may help to elucidate the potential signaling pathways of these stresses and the roles of LRRC45 and CCDC91 in this context.

## Supplementary Material

bpaf048_Supplementary_Data

## Data Availability

Data not publicly available.
