# Supplementary material for: Novel reporter systems to detect cold and osmotic stress responses
Source: Biol Methods Protoc. 2025 Jun 14;10(1):bpaf048. doi: 10.1093/biomethods/bpaf048 (PMC12206525; doi:10.1093/biomethods/bpaf048)
Supplement: bpaf048_Supplementary_Data [file bpaf048_supplementary_data.zip › Supplementary_Data.zip!/Maruyama & Fujii_Supplementary info.pdf]

Novel reporter systems to detect cold and osmotic stress responses

Kanon Maruyama[1], Hodaka Fujii[1]*

[1]Department of Biochemistry and Genome Biology, Hirosaki University Graduate School of Medicine, 5 Zaifu-cho, Hirosaki, Aomori 036-8562, Japan

Department of Biochemistry and Genome Biology, Hirosaki University Graduate School of Medicine, 5 Zaifu-cho, Hirosaki, Aomori 036-8562, Japan

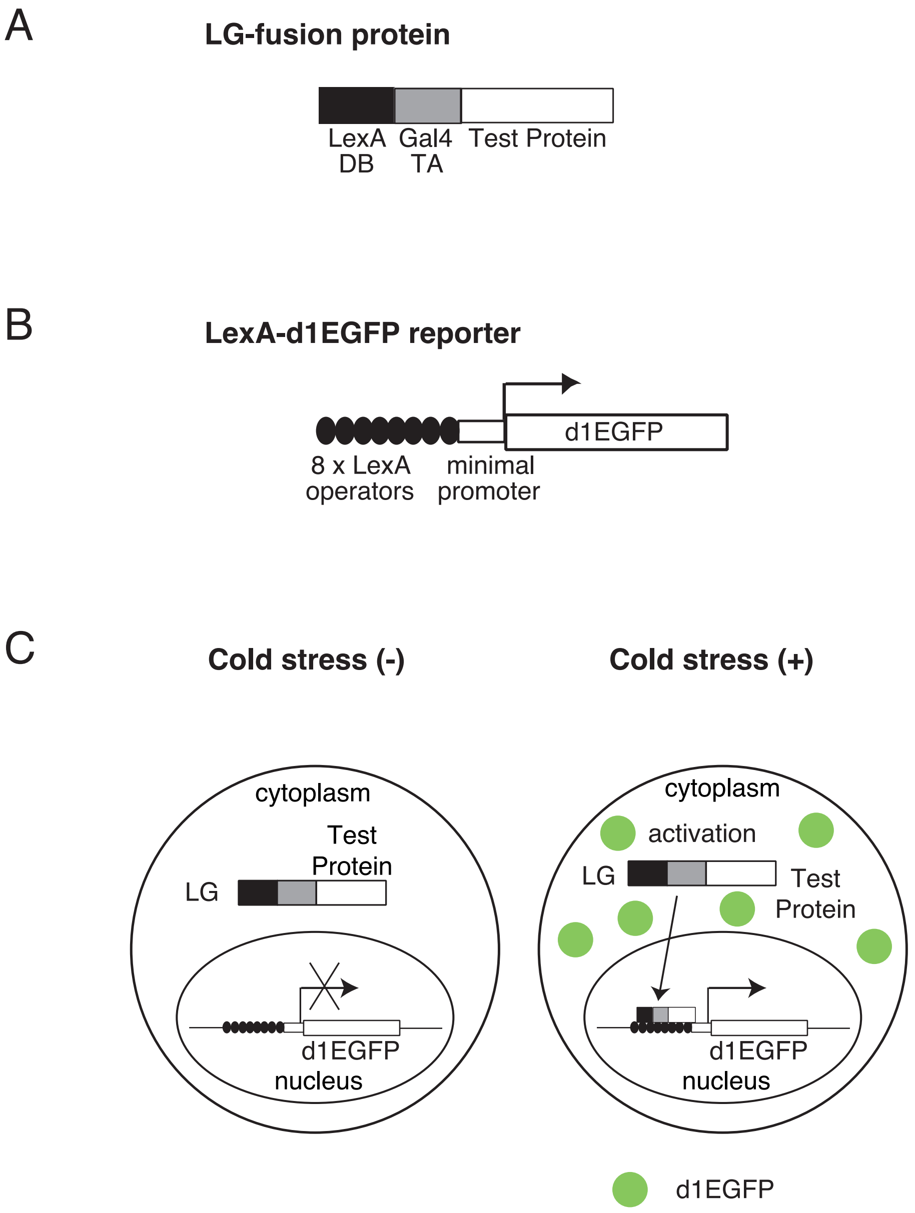

Supplementary Fig. S1. The ITT system to detect cold stress-induced nuclear translocation. (A) A LG-fusion protein consisting of LexA DB, the TA domain of Gal4, and a test protein. **(B)** The LexA-d1EGFP reporter gene consisting of 8 × LexA operators, a minimal promoter, and d1EGFP. (**C**) A scheme of d1EGFP expression induced by nuclear translocation of the LG-fusion protein. When the LG-fusion protein is in the cytoplasm, d1EGFP is not expressed. When cold stress induces nuclear translocation of the fusion protein, it binds to the LexA operators via LexA DB and activates d1EGFP expression via the action of the TA domain of Gal4.

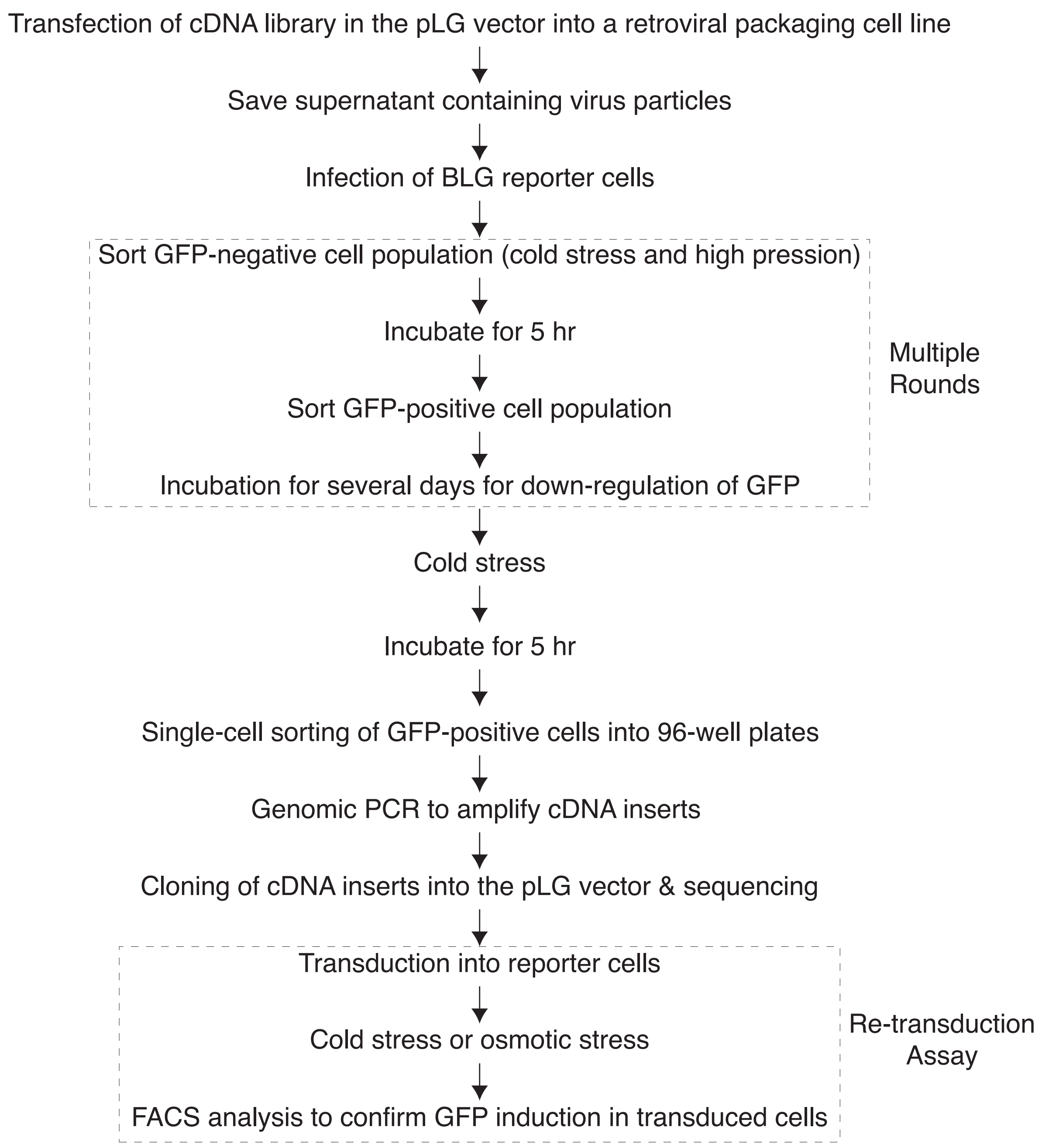

Supplementary Fig. S2. Scheme of screening of cDNA libraries for stress-induced reporter activation.
